# Neurilemmoma of Retromolar Region in the Oral Cavity

**DOI:** 10.1155/2015/320830

**Published:** 2015-06-29

**Authors:** Ajit Singh Rathore, Deepti Srivastava, Nidhi Narwal, Devi Charan Shetty

**Affiliations:** Department of Oral Pathology, I.T.S. Dental College, Murad Nagar, Ghaziabad, Uttar Pradesh 201206, India

## Abstract

Neurilemmoma also known as schwannoma is benign nerve sheath tumor rarely occurring in the oral cavity. Only 1% of all extracranial schwannomas show that intraoral occurrence with tongue is the commonest site and retromolar region is the least common site. It presents as encapsulated, slow growing, solitary, smooth-surfaced, usually asymptomatic tumor. We report a case of 70-year-old male with well-defined mass on left retromolar region which was painless and slow growing. Diagnosis is made by histological examination and immunohistochemistry analysis to confirm the neural tissue origin of the lesion. The treatment is complete surgical excision of the lesion without recurrence.

## 1. Introduction

Schwann cells form a thin outline around each extracranial nerve fiber and wrap larger fibers with an insulating membrane, myelin sheath, to enhance nerve conductance. Schwannomas arise when proliferating Schwann cells form a tumor mass encompassing motor and sensory peripheral nerves [1].

So, schwannoma, also called neurilemmoma, neurinoma, and perineural fibroblastoma, is a solitary, benign, encapsulated, and slow growing tumor, arising from neural sheath's Schwann cells of the peripheral, cranial (except for the optic and olfactory), spinal, and autonomic nerves [1, 2]. These tumors have a predilection for the head and neck, the extremities, and the posterior mediastinum [3]. In the head and neck area, they primarily appear in soft tissue and often in the tongue, and intraosseous schwannomas are particularly rare, making up less than 1% of all benign primary bone tumors [2, 3].

Size and locations of lesions determine the presence and intensity of symptoms [1]. Schwannomas are usually solitary lesions but in unusual instance are multiple or occur in the setting of von Recklinghausen's neurofibromatosis [1, 4].

It was first described by Verocay in 1910 [5]. It is relatively an uncommon neural neoplasm with unknown etiology [4]. Malignant transformation is rare and incidence of malignant schwannomas ranges from 8% to 13.9% [6]. Neurilemmoma is usually seen in third and fourth decade of life. We hereby present a case report of neurilemmoma of retromolar region in a 70-year-old male through imaging, morphological, and immunohistochemical studies.

## 2. Case Report

**Figure 1:**
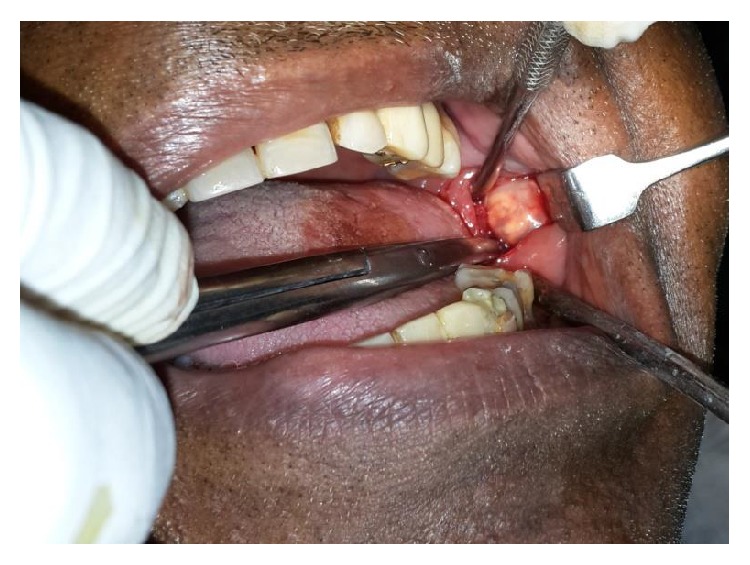
Asymptomatic nodule in left retromolar region.

**Figure 2:**
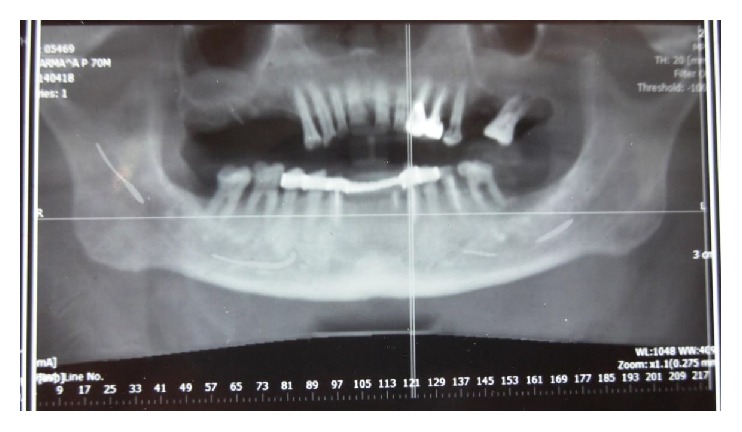
CBCT panorama view reveals heterogeneous soft tissue mass in left retromolar region.

**Figure 3:**
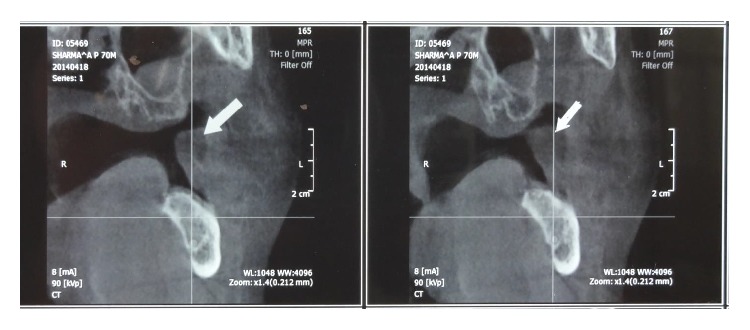
CBCT reveals heterogeneous lesion in left buccomasseteric region extending into left sided retromolar region.

A 70-year-old male patient presented with complaint of swelling in lower left back tooth region for 1 year. The swelling was gradually increasing in size. On clinical examination a 3 × 3 cm sized mass was present on left retromolar region of normal mucosal colour (Figure 1), smooth surface, soft in consistency, tender on palpation, and relatively mobile. Rest of the oral cavity was normal. His general condition and nutritional status were good. The patient was otherwise healthy and had no significant past medical history and no other symptoms; his family history was also unremarkable. Clinically the lesion appeared to be benign soft tissue neoplasm and provisional diagnosis of intraoral lipoma was suggested. The complete blood count gave results within normal limits. Cone beam computer tomography revealed heterogeneous lesion in left buccomasseteric region extending into left sided retromolar region (Figures 2 and 3).

**Figure 4:**
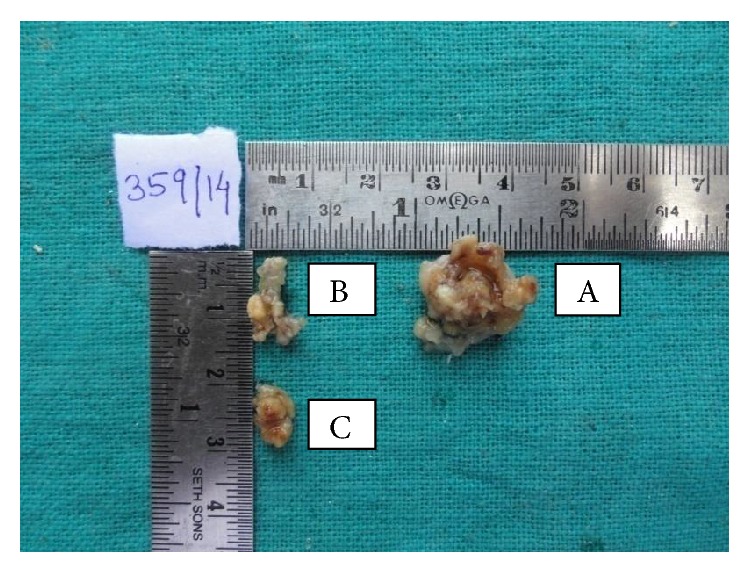
Gross specimen showing asymmetrical yellow colored 3 soft tissue bits received (A: 1.5 × 1.5 cm, B: 0.9 × 0.5 cm, and C: 0.6 × 0.9).

Excision of the mass was done with adequate surgical margins of resection. Biopsy specimen was sent for histopathological examination. Macroscopically, one bit is large in size 1.5 × 1.5 cm, oval in shape, and yellowish in color and has irregular surface. The other two bits are 0.9 × 0.5 cm and 0.6 × 0.9 cm in size, respectively (Figure 4).

**Figure 5:**
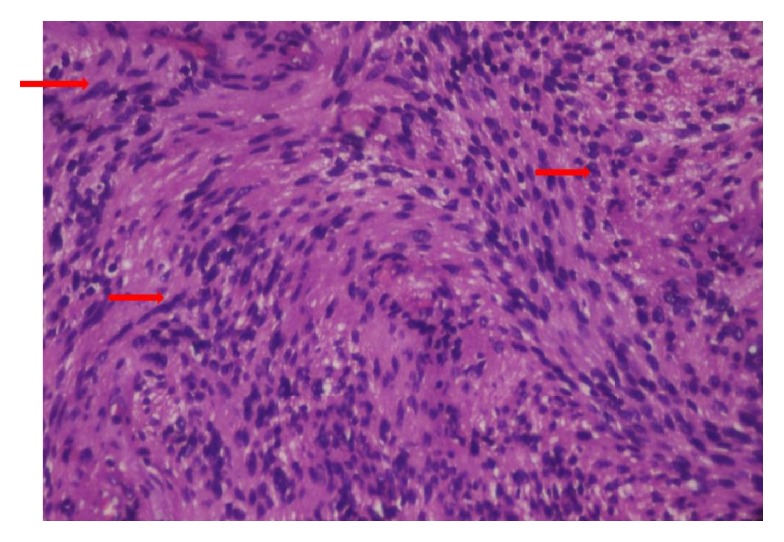
Photomicrograph showing spindle cells with wavy nuclei to round to ovoid nuclei indistinct cytoplasmic borders arranged in interlacing fascicles (H&E 40x).

**Figure 6:**
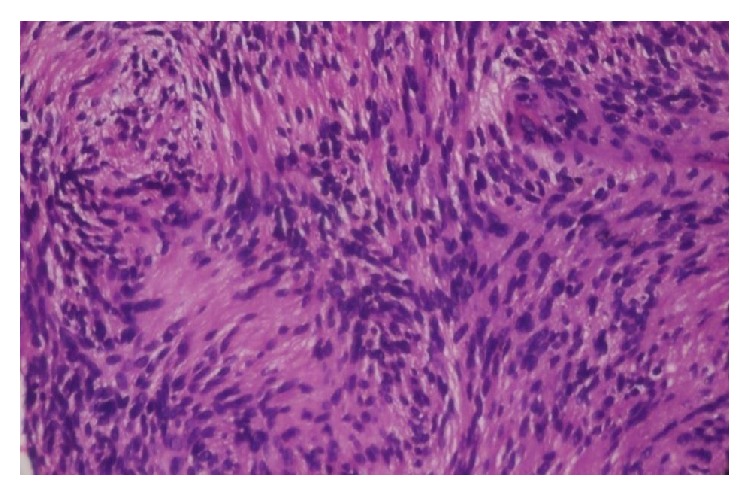
Photomicrograph showing nuclear palisading with Verocay bodies (Antoni A areas) (H&E 40x).

**Figure 7:**
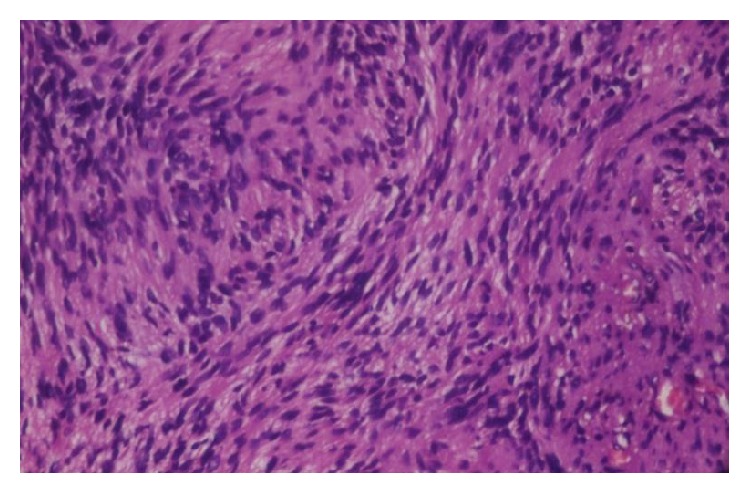
Photomicrograph showing spindle or oval cells in loosely textured matrix (Antoni B areas) (H&E 40x).

**Figure 8:**
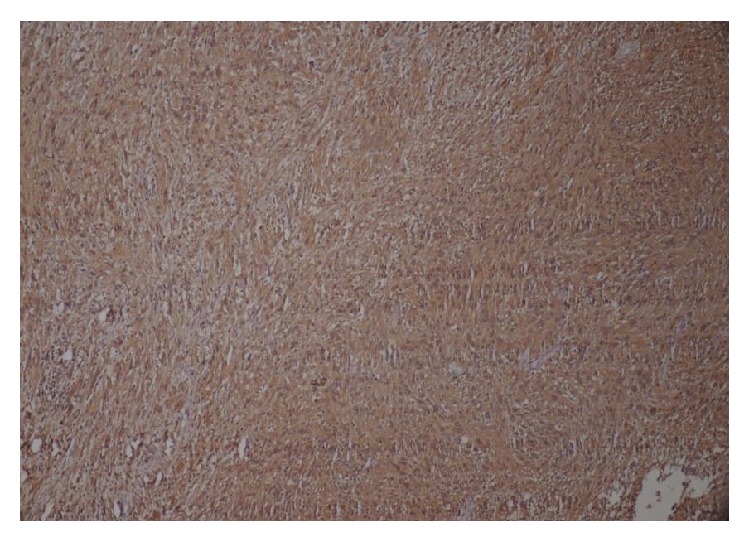
Schwann cells showing strong and diffuse positivity of S-100.

Microscopically the tissue showed highly cellular areas enclosed within a fibrous capsule. The tumor was composed of fascicular pattern predominantly with nuclear palisading appearing like Antoni A areas (Figure 5) with intermittent homogeneous eosinophilic areas resembling Verocay bodies (Figure 6). Cellular components were spindle, round to ovoid shape cells with plump and hyperchromatic nuclei and focal areas of pleomorphic cells were also seen. Some ovoid cells exhibited blunt ended nuclei. Focal areas of Antoni B type hypocellular areas were composed of spindle and oval cells arranged haphazardly in loosely textured matrix which also showed few inflammatory cells and delicate collagen fibres (Figure 7). Few thick walled blood vessels and adipocytes were seen. Nerve bundles were also appreciated in the fibrous capsule. Vimentin, smooth muscle actin, and S-100 protein (Figure 8) were performed where S-100 protein and vimentin were found to be strongly reactive in the spindle cells while smooth muscle actin was negative. Histopathological diagnosis of neurilemmoma was given.

## 3. Discussion

Schwannomas are benign encapsulated nerve sheath neoplasm composed of Schwann cells [7]. Wright and Jackson reported 146 cases of schwannoma of the oral cavity soft tissue. Of those, 52% occurred in the tongue, 19.86% in the buccal or vestibular mucosa, 8.9% in the soft palate, and the remainder 19.24% in the gingivae and lip [8].

Embryologically, Schwann cells arise during the fourth week of development from a specialized population of ectomesenchymal cells derived from neural crest. These cells serve as thin barrier around each extracranial nerve fiber of motor and sensory nerves and wrap larger fibers with myelin sheath to enhance nerve conductance [9]. Schwannomas commonly arise from spinal nerve roots and intracranial nerves of the face, neck, extremities, mediastinum, and pelvis. Most commonly affected nerve is the VIII cranial nerve (acoustic neuromas) [7, 9].

Diagnostic investigations include ultrasound scanning, computed tomography, MRI, and fine-needle aspiration cytology. Ordinary histopathology provides definitive diagnosis of schwannoma. The tumor tissue consists of the so-called Antoni A and B type cells. Type A areas show densely packed, elongated spindle cells with nuclear palisading around central acellular eosinophilic areas representing Verocay bodies formed by thin cytoplasmic fibers and reduplicated basement membrane while type B tissue has a more myxoid consistency. Additionally, hemorrhage from adjacent tissue, necrosis, hyalinization, and cystic degeneration may also occur in the tumor tissue [10].

The main clinical differential diagnoses consisting of other benign neoplasms at this site include neurofibroma, traumatic neuroma, nonossifying fibroma, lipoma, and leiomyoma [10]. The schwannomas may be indistinguishable from other benign tumors, so biopsy and histological examination are essential.

Tumors histologically simulating neurilemmomas in this case are leiomyoma, well-differentiated leiomyosarcoma, fibrosarcoma, and malignant peripheral nerve sheath tumor.

Neurofibroma may be a manifestation of neurofibromatosis type 1 (NF-1) and the probability of recurrence in neurofibroma is higher than schwannoma. Neurofibroma has the potential for malignant transformation and about 15-16% of patients with neurofibromatosis present with malignant transformation. Neurofibromas lack the thick collagenous capsule of schwannomas.

With lipoma, superficial varieties are easily recognized by their softness and yellow color, which is usually overlaid with a clear pattern of minuscule arterial blood vessels. Lipomas are rare in the oral cavity. Nonossifying fibroma often occurs in young people less than 20 years old, clinically with less pain [11].

Malignant transformation of schwannomas in the head and neck region is unusual. Das Gupta and Brasfield notified an incidence of 8% of malignant schwannomas and Ghosh et al. reported an incidence of 13.9% [12, 13]. Treatment of schwannoma is complete surgical excision of the lesion which does not result in any recurrence [14].

## 4. Conclusion

Few cases of intraoral neurilemmoma of retromolar region have been reported. The histopathological features are characteristic for diagnosis of the neurilemmoma but few cases may pose diagnostic dilemma owing to cellular characteristic as seen in the present case. Therefore, immunohistochemical analysis of panel of markers with their consistent expression and intensity may serve as important diagnostic tool.
